# Complications and outcomes after definitive surgery for traumatic high-energy pelvic fractures

**DOI:** 10.1007/s00590-026-04729-7

**Published:** 2026-03-30

**Authors:** Kaarina Birgitta Salonpää, Juho Nurkkala, Ulla Koskela, Iikka Lantto, Sanna Kakko, Timo Kaakinen, Janne Liisanantti

**Affiliations:** 1 University of Oulu, Research Unit of Translational Medicine, Oulu, Finland; 2 Helsinki University Hospital, Helsinki, Finland

**Keywords:** Pelvic fractures, High-energy trauma, Definitive surgery, Postoperative complications

## Abstract

**Background:**

Postoperative complications and prolonged recovery are often recorded in traumatic high-energy pelvic fractures. This study aimed to assess the rate of postoperative complications and identify factors associated with their occurrence following surgical treatment.

**Methods:**

This retrospective study included 162 patients with traumatic high-energy pelvic fractures who underwent surgery at Oulu University Hospital between 2010 and 2021. Patient demographics, laboratory findings, operative details, and postoperative outcomes were reviewed.

**Results:**

A total of 68 postoperative complications were recorded, comprising 34 surgical and 34 medical events. Patients who developed complications were older, had higher C-reactive protein (CRP) levels on admission, and longer operative times compared with those without complications. No significant difference in the time from hospital admission to surgery was observed between the groups.

**Conclusion:**

Postoperative complications following traumatic pelvic fractures were common. Older age was independently associated with an increased risk of postoperative complications.

**Supplementary Information:**

The online version contains supplementary material available at 10.1007/s00590-026-04729-7.

## Introduction

High-energy pelvic fractures are associated with a high rate of postoperative complications and prolonged in-hospital recovery [1, 2]. In high-energy trauma, up to 25% of patients sustain pelvic fractures [3]. The most common injury mechanism is a high fall, accounting for roughly half of all cases, while road traffic accidents make up the majority of the remaining injuries [1]. In Europe, the incidence of high-energy pelvic fractures is increasing for reasons that remain unclear, and the treatment trend is increasingly surgical [4]. Besides the significant economic burden these severe injuries impose, pelvic fractures often lead to substantial functional impairment and negatively impact long-term quality of life [5–7].

Nearly one-third of patients with high-energy pelvic fractures experience at least one postoperative complication [3]. Previous studies have reported unplanned reoperation rates ranging from 15% to 22%. Among medical complications, pneumonia is the most common after emergency pelvic surgery [8]. Other common medical complications include thromboembolic events, renal and cardiac failure, and various infections [3, 8, 9]. However, factors that predispose patients to early postoperative complications after pelvic surgery are only partially understood [3, 9]. Prior reports often lack detailed information about when complications occur [2, 3, 8–10]. This study aims to investigate complication rates and clinical outcomes following surgery for high-energy pelvic fractures. We hypothesize that advanced age, elevated inflammatory status, and perioperative factors are associated with postoperative complications.

## Methods

### Patients

This was a retrospective single-center study including 162 adult patients with traumatic high-energy pelvic fractures who underwent surgery at Oulu University Hospital. Oulu University Hospital is a tertiary-level trauma center in northern Finland, serving a population of approximately 700,000 inhabitants. Patients who underwent reoperation were recorded only once. All surgically treated pelvic fractures between January 1, 2010, and April 15, 2021, were identified from the hospital’s surgical database using ICD-10 (S32.1 sacral fracture, S32.4 acetabulum fracture, S32.7 multiple pelvic ring fractures and m84.3 stress.

fracture) and operation codes (NEJ50 pelvic ring fixation, NEJ60 acetabulum fracture fixation, NFB60 hard total hip replacement and NEJ86 pelvic fracture re- or late fixation. Motor vehicle accidents and high falls causing pelvic fractures were considered as high-energy injuries, whereas low-energy mechanisms of injury were excluded in accordance with previous literature [11]. Complications were classified as medical or surgical, consistent with our previous study [12]. Only in-hospital postoperative complications were recorded, and the primary outcome in this study was to analyze and report postoperative complications after definitive surgery for traumatic high-energy pelvic fractures.

### Data extraction

Data were manually extracted from the medical records. Collected variables included age, gender, weight, and history of smoking and alcohol use. Chronic health status was assessed using the Charlson Comorbidity Index (CCI) and documented diagnoses. Vital signs, including blood pressure, oxygen saturation, and Glasgow Coma Scale (GCS), were recorded during prehospital care, in the emergency department, throughout the intraoperative phase, and during postoperative care. Intraoperative blood loss and the need for blood products during hospitalization were obtained from the patient data management system. Blood samples, including hemoglobin and C-reactive protein (CRP), were collected at admission and on the day of surgery during the laboratory’s morning round between 6 and 8 am.

### Statistical analysis

IBM SPSS Statistics 29 software (IBM SPSS Statistics for Windows, Version 29.0, Armonk, NY, USA) was used to conduct statistical analyses. Categorical variables are presented as numbers (n) and percentages (%), while continuous variables are shown as medians with 25–75th percentiles [25–75th PCT]. Categorical variables were analyzed using Pearson’s chi-square test, and continuous variables were examined with the Mann–Whitney test. Two-tailed P values below 0.05 were considered statistically significant. Logistic regression analysis was performed to calculate the OR for postoperative complications. Age, gender, and both continuous and categorical variables with univariate significance < 0.1 were included one by one using the enter method. Factors significantly impacting the log-likelihood function were retained in the model, while those without positive influence were excluded to prevent model overfitting.

## Results

A total of 162 patients with high-energy pelvic fractures underwent surgical treatment during the study period. Acetabular fractures were documented in 58 patients, while the remaining 104 patients had pelvic fractures. The types of injuries are shown in Tables 1 and 2. The Modified Stoppa approach was the most common procedure used, and only two patients received external fixation (Table 3). Overall, 68 postoperative complications were recorded in 46 patients (28.4%), with 34 classified as surgical complications and 34 as medical complications. The most common surgical complication was surgical site infection (SSI), which occurred in 12 cases (17.6%), usually on the ninth postoperative day. Pneumonia was the most common medical complication, affecting 17 cases (25.0%). There were 17 reoperations in this group (Table 4).

Patients with postoperative complications were older, had lower admission hemoglobin levels, received blood products more frequently during admission, had higher admission AIS and ISS scores, and experienced longer durations of surgery compared to patients without postoperative complications. The time between injury and surgery was similar in both groups (6 [3–9] vs. 6 [3–8], *P* = 0.264). No other variables differed significantly between patients with and without postoperative complications (Table 5).

In the logistic regression analysis, age over 60 years and elevated CRP levels on the day of operation were identified as risk factors for postoperative complications (Table 6).

Patients with postoperative complications had longer hospital LOS (16 [12–21] vs. 10 [6–15], *P* < 0.001). There were no differences in survival or discharge location (Table 7).

## Discussion

Overall, 68 postoperative complications were recorded in 46 patients (28.4%), with 34 classified as surgical and 34 as medical complications. The observed rate was consistent with previously published data [1, 3]. Surgical and medical complications occurred at similar rates, with 34 cases in each group. Seventeen patients needed reoperation during their hospital stay. The most common surgical complication was surgical site infection (SSI), and the median time to reoperation was 11 days after the initial surgery. One patient died during hospitalization.

Pneumonia was the most common medical complication. It occurred in 17 patients (10.4%), consistent with earlier reports, which indicated rates ranging from 7% to 13% among surgically treated high-energy pelvic trauma patients [8, 9]. In pelvic trauma, chest injuries are the most frequent associated injury pattern; over one-third of such patients experience rib fractures or pulmonary contusions [8]. Thoracic trauma has been independently associated with an increased risk of pneumonia in polytrauma patients, and postoperative pneumonia is recognized as an independent predictor of mortality [13, 14]. Although the risk of postoperative pneumonia is well known, its onset has been poorly documented in this specific patient population. In our cohort, pneumonia usually developed during the early postoperative period. This emphasizes the importance of vigilant postoperative monitoring, early detection of respiratory decline, and comprehensive, multimodal care, including lung-protective ventilation strategies and appropriately targeted antimicrobial therapy.

SSIs accounted for over one third (35.3%) of all surgical complications in this study. This proportion was slightly lower than that reported in recent comparable studies [3, 9]. Although SSI occurrence is well documented in the literature [2, 3, 9, 15], the timing of onset has rarely been described in detail. In our series, SSIs typically manifested more than one week after the initial operation. This delayed onset presents a particular clinical challenge: unlike other surgical complications, such as bleeding or fixation failure, SSIs may develop silently, leading to significant delays in recovery. This highlights the importance of remaining vigilant for the initial signs of infection following pelvic surgery.

In the logistic regression analysis, age over 60 years and elevated C-reactive protein (CRP) levels on the day of surgery were independently associated with postoperative complications. Although advanced age has traditionally been linked to a higher risk of postoperative morbidity [8], some recent studies have reported conflicting findings regarding acetabular and pelvic fractures. For instance, Lundin et al. found no association between age > 60 years and adverse events or reoperation rates after acetabular fracture surgery [9]. Similarly, Mostert et al. reported no correlation between age and SSI in patients undergoing pelvic fracture fixation, most of whom had sustained high-energy trauma [3]. It should be noted, however, that the median age in those studies was higher than in our cohort, which may have reduced the observed effect of age on complication risk. When interpreting our results, it is also important to consider that younger patients are typically involved in higher-energy mechanisms of injury [8]. Since our study included exclusively high-energy trauma cases, this might explain both the relatively young median age and the association between older age and increased complication rates. Preoperative inflammation, as indicated by elevated CRP levels, has previously been associated with postoperative morbidity and complications in various surgical disciplines [16–18]. Therefore, the observed association between elevated CRP on the day of surgery and postoperative complications was expected, though previously underreported, in the context of high-energy pelvic fractures. Based on these findings, one could hypothesize that preoperative CRP assessment may help identify high-risk patients who could benefit from intensified perioperative monitoring and early preventive measures.

The optimal timing for definitive surgical fixation in pelvic fractures remains controversial. The definition of early surgery varies widely in the literature, ranging from within eight hours to up to one week after injury [7, 15]. Dormann et al., who conducted the only systematic review on the timing of definitive fixation for pelvic fractures in polytrauma patients, included only one study due to the limited number of eligible studies, making the overall evidence insufficient [19]. A more recent study that combined pelvic and acetabular fracture cases found no difference in outcomes between early and delayed surgery (using 72 h as the cutoff), although intraoperative blood loss was higher in the early group [15]. In our study, no significant association was found between the timing of definitive fixation and postoperative complications, nor was there a difference between procedures performed within 72 h versus after 72 h from injury. However, the interval from injury to definitive surgery was relatively long compared to other reports [15, 19]. This likely reflects the need for initial stabilization and management of coexisting parenchymal or orthopedic injuries frequently seen in patients with high-energy trauma [3, 9, 10].

Patients who developed postoperative complications had longer hospital stays, but no other statistically significant associations between complications and clinical outcomes were observed. About one-third of patients in our study were discharged directly home, which aligns with previous findings [11]. Interestingly, postoperative complications did not seem to affect the discharge destination. One might expect that patients with complications would more often need transfer to step-down care in regional hospitals or community health centers. It is well known that pelvic fractures significantly impair physical function and quality of life during early recovery, which likely explains the relatively low rate of direct home discharge [5, 6].

## Limitations

This study has several limitations. Although data were collected over an extended period, the sample size was small and limited to a single center, thereby reducing statistical power and the generalizability of the findings. High-energy pelvic fractures are relatively rare, accounting for only 1%–3% of all skeletal injuries [4, 11], making data collection challenging. Additionally, there is significant heterogeneity in both pelvic injury types and potential surgical treatment options, which complicates the interpretation of the results. Nevertheless, our sample size was comparable to that of other published studies [1, 3, 9]. While elevated CRP was associated with postoperative complications, the current study design does not allow us to determine whether it reflects systemic inflammation, early infection, or injury severity, which is a limitation. The retrospective nature of the study inherently limits causal conclusions, although logistic regression was used to reduce confounding. Patient-reported outcomes were not collected, and only in-hospital outcomes were examined, potentially underestimating the true complication rate.

## Conclusion

Nearly one-third of patients experienced at least one postoperative complication. Advanced age and elevated inflammatory markers were associated with the occurrence of postoperative complications. Patients who developed complications had longer hospital stays, but no other significant associations with clinical outcomes were found. The timing of definitive fixation did not seem to affect the occurrence of postoperative complications.

## Supplementary Information

Below is the link to the electronic supplementary material.

Supplementary Material 1

## Data Availability

No datasets were generated or analysed during the current study.
