# Supplementary material for: Complications and outcomes after definitive surgery for traumatic high-energy pelvic fractures
Source: Eur J Orthop Surg Traumatol. 2026 Mar 30;36(1):151. doi: 10.1007/s00590-026-04729-7 (PMC13035693; doi:10.1007/s00590-026-04729-7)
Supplement: Supplementary file 1 — Supplementary Material 1 [file 590_2026_4729_MOESM1_ESM.docx]

Table 1. Letournel classification of 58 acetabulum fracture patients with and without postoperative complications

|  | Patients with postoperative complications n=14 | Patients without postoperative complications n=44 | p-value |
| --- | --- | --- | --- |
| AC | 1 | 5 | 0.313 |
| AC+PW | 0 | 1 |  |
| ACPHT | 1 | 2 |  |
| BC | 6 | 3 |  |
| PC | 0 | 2 |  |
| PC+PW | 1 | 4 |  |
| PW | 2 | 14 |  |
| T-shape | 2 | 3 |  |
| Transverse | 1 | 6 |  |
| Transverse+AW | 0 | 1 |  |
| Transverse+PW | 0 | 2 |  |
| Unclassified | 0 | 1 |  |

AC; Anterior Column, PW; Posterior Wall, ACPHT; Anterior Column Posterior Hemitransverse, BC; Both Columns, PC; Posterior Column, AW; Anterior Wall

Table 2. Young-Burgess classification of 104 pelvic fracture patients with and without postoperative complications

|  | Patients with postoperative complications n=31 | Patients without postoperative complications n=73 | p-value |
| --- | --- | --- | --- |
| VS | 16 (51.6) | 25 (34.2) | 0.475 |
| LC1 | 6 (19.4) | 28 (38.4) |  |
| LC2 | 4 (12.9) | 6 (8.2) |  |
| LC3 | 0 (0.0) | 1 (1.4) |  |
| APC1 | 1 (3.2) | 2 (2.7) |  |
| APC2 | 2 (6.5) | 8 (10.0) |  |
| APC3 | 1 (3.2) | 1 (1.4) |  |
| CM | 1 (3.2) | 0 (0.0) |  |
| Unclassified | 0 (0.0) | 1 (1.4) |  |

VS; Vertical Shear, LC; Lateral Compression, APC; Anterior Posterior Compression, CM; Combined Mechanism

Table 3. Types of surgical approaches

|  | Patients with postoperative complications n=46 | Patients without postoperative complications n=116 | p-value |
| --- | --- | --- | --- |
| Transsacral screws | 6 (13.0) | 13 (11.2) | 0.214 |
| Spinopelvic fixation | 1 (2.2) | 4 (3.4) |  |
| SI-screw | 5 (10.9) | 25 (21.6) |  |
| Kocher-Langenbeck | 6 (13.0) | 30 (25.9) |  |
| Iliac Window | 2 (4.3) | 6 (5.2) |  |
| External Fixation | 1 (2.2) | 1 (0.9) |  |
| Modified Stoppa | 23 (50.0) | 34 (29.3) |  |
| Unclassified | 2 (4.3) | 3 (2.6) |  |

Table 4. Postoperative complications of 162 trauma patients with high energy pelvic fracture

|  | Number of complications n=68 | Complication onset (days from operation) |
| --- | --- | --- |
| Surgical complications | 34 (50.0) | 9 [2-16] |
| Iatrogenic vessel trauma | 4 (5.9) | NA |
| Postoperative bleeding | 1 (1.6) | 1 |
| Surgical site infection | 12 (17.6) | 9 [3-16] |
| Reoperation | 17 (25.0) | 11 [5-21] |
|  | | |
| Medical complications | 34 (50.0) | 3 [1-4] |
| AKI | 5 (7.4) | 1,1,1,3,9 |
| Pneumonia | 17 (25.0) | 2 [1-3] |
| Deep venous thrombosis | 4 (5.9) | 3,20,21,24 |
| Pulmonary embolism | 6 (8.8) | 1,1,3,3,4,19 |
| Cardiologic complication | 2 (2.9) | 1,2 |

AKI; Acute kidney injury.

Table 5. Comparison of baseline characteristics and injuries

|  | Patients with postoperative complications n=46 (28.4) | Patients without postoperative complications n=116 (71.6) | P-value |
| --- | --- | --- | --- |
| Male | 26 (56.5) | 79 (68.1) | 0.164 |
| Age (years) | 50 [34-71] | 45 [28-57] | 0.072 |
| Age >60 years | 17 (37.0) | 24 (20.7) | 0.026 |
| BMI | 26.2 [22.0-29.5] | 25.1 [21.9-28.3] | 0.461 |
| Smoking | 12 (26.1) | 23 (19.8) | 0.383 |
| Alcohol abuse | 7 (15.2) | 18 (15.5) | 0.962 |
| Charlson comorbidity index | 1 [0-4] | 0 [0-2] | 0.080 |
| From injury to operation (days) | 6 [3-9] | 6 [3-8] | 0.264 |
| From injury to operation ≥72h | 32 (69.6) | 81 (69.8) | 0.834 |
| CRP on the day of surgery | 77 [47-163] | 61 [23-118] | 0.068 |
| Admission Hb | 116 [109-129] | 127 [114-137] | 0.006 |
| Blood/platelet transfusion at the beginning of admission | 13 (28.3) | 18 (15.5) | 0.031 |
| Intraoperative blood loss (mL) | 400 [100-900] | 300 [100-550] | 0.291 |
| Admission ISS score | 17 [13-30] | 16 [9-25] | 0.041 |
| Admission ISS>15 | 34 (73.9) | 64 (55.2) | 0.028 |
| Admission AIS score | 5 [4-9] | 5 [3-8] | 0.059 |
| Operation duration (min) | 188 [134-268] | 143 [73-191] | 0.006 |
| Acetabulm fracture | 14 (30.4) | 44 (37.9) | 0.449 |
| Parenchymal trauma | 25 (54.3) | 55 (47.4.) | 0.426 |
| Associated ortopedic injury (open fracture, long bone trauma, spine trauma) | 26 (56.5) | 64 (55.2) | 0.876 |

BMI; Body mass index, CRP; C-reactive protein, Hb; Hemoglobin, ISS; injury severity score, AIS; Abbreviated injury scale

Table 6. Logistic regression analysis for postoperative complications

|  | OR and 95% CI’s | p-value |
| --- | --- | --- |
| Age>60 years | 4.77 [1.63-13.96] | 0.004 |
| CRP on the day of surgery | 1.01 [1.00-1.02] | 0.038 |
| Admission Hb | 0.978 [0.955-1.001] | 0.062 |

OR; Odds ratio, CI; Confidence interval, CRP; C-reactive protein, Hb; Hemoglobin

Table 7. Outcomes

|  | Patients with postoperative complications n=46 | Patients without postoperative complications n=116 | P-value |
| --- | --- | --- | --- |
| Reoperation | 17 (37.0) | 0 (0.0) | <0.001 |
| ICU admission | 32 (69.6) | 62 (53.5) | 0.061 |
| Hospital LOS | 16 [12-21] | 10 [6-15] | <0.001 |
| Three-month mortality | 1 (2.2) | 0 | 0.092 |
| Survival (days) | 1726 [1629--] | 2432 [1430--] | 0.800 |
| Discharged home | 11 (23.9) | 38 (32.8) | 0.269 |
| Discharged to regional health care center’s ward | 13 (28.3) | 29 (25.0) | 0.669 |
| Discharged regional hospital | 21 (45.7) | 49 (42.2) | 0.693 |

ICU; intensive care unit, LOS; Length of stay
